# A standardized herbal combination of *Astragalus membranaceus* and *Paeonia japonica* promotes skeletal muscle hypertrophy in a treadmill exercise mouse model

**DOI:** 10.3389/fnut.2024.1362550

**Published:** 2024-06-20

**Authors:** Sung-Bae Lee, Tae-Wook Woo, Dong-Cheol Baek, Chang-Gue Son

**Affiliations:** Institute of Traditional Medicine and Bioscience, Daejeon University, Daejeon, Republic of Korea

**Keywords:** skeletal muscle, hypertrophy, *Astragalus membranaceus*, *Paeonia japonica*, exercise

## Abstract

**Background:**

Maintaining a normal range of muscle mass and function is crucial not only for sustaining a healthy life but also for preventing various disorders. Numerous nutritional or natural resources are being explored for their potential muscle hypertrophic properties.

**Aim:**

We aimed to evaluate the muscle hypertrophic effects of APX, a 1:1 mixture of *Astragalus membranaceus* and *Paeonia japonica*. In addition to the myotube differentiation cell assay, we utilized a weighted exercise-based animal model and evaluated changes in muscle hypertrophy using dual-energy X-ray absorptiometry (DXA) and histological analysis.

**Results:**

The 8-week treadmill exercise led to notable decreases in body weight and fat mass but an increase in muscle mass compared to the control group. Administration of APX significantly accelerated muscle mass gain (*p* < 0.05) without altering body weight or fat mass compared to the exercise-only group. This muscle hypertrophic effect of APX was consistent with the histologic size of muscle fibers in the gastrocnemius (*p* > 0.05) and rectus femoris (*p* < 0.05), as well as the regulation of myogenic transcription factors (MyoD and myogenin), respectively. Furthermore, APX demonstrated a similar action to insulin-like growth factor 1, influencing the proliferation of C2C12 myoblast cells (*p* < 0.01) and their differentiation into myotubes (*p* < 0.05) compared to the control group.

**Conclusion:**

The present study provides experimental evidence that APX has muscle hypertrophic effects, and its underlying mechanisms would involve the modulation of MyoD and myogenin.

## 1 Introduction

In the human body, skeletal muscle is a major tissue accounting for 35% of body weight (1), that plays key roles in movement, maintaining posture and temperature and stabilizing joints (2). Skeletal muscle also supplies essentials such as amino acids or glucose to the brain, heart, and liver in the case of a lack of nutrients (3, 4). In general, muscle function tends to decrease by approximately 50% in individuals in their 80s compared to their peak in their 20s, due to age-related reductions in muscle mass, strength, and contraction (5, 6). This reduction of muscle function is directly related to high incidence of type 2 diabetes and osteoporosis (7, 8). In certain diseases, including heart diseases and cancer, the continuous reduction in muscle mass causes sarcopenia, leading to high mortality (9, 10). Therefore, maintaining the normal range of muscle mass and function is needed not only to maintain a healthy life but also to prevent various disorders (11).

For a healthy life, many people perform physical activities, known as exercise, such as jogging, yoga or weight training, and these exercise markets have grown 3.5-fold over the past 5 years (12). The beneficial effects of exercise including personal physical development, psychological well-being and reduced risk of chronic diseases, have been reported over the course of years of research (13–15). Compared to mere exercise, resistance exercise has been shown to be even more effective for muscle hypertrophy (16). A systematic analysis has revealed that the positive effects of exercise are further enhanced when combined with dietary supplements such as proteins (17). Many individuals have begun incorporating dietary supplements into their routines, including not only proteins but also amino acids, vitamins, and other organic compounds (18). Regarding muscle hypertrophy, these supplements are thought to involve various mechanisms, including protein synthesis, energy metabolism, oxidative stress, inflammation, and/or muscle damage during exercise (19). Therefore, the dietary supplement industry is currently a large market, with a gross of USD 72 billion in 2021 (20), while concerns about the real efficacy and safety of dietary supplements remain due to the lack of scientific evidence (21). In fact, it was even reported in 2020 that an excessive intake of vitamins or minerals is related to a high risk of chronic diseases, including cardiovascular disease, type 2 diabetes, osteoporosis and cancer (22).

On the other hand, herbal resources have been widely utilized to provide health-related benefits for both the general population and diseased individuals, drawing from extensive clinical experience. Some medicinal herbs have been reported to enhance muscle strength, lean body mass, or performance. For instance, ginseng has been associated with improved endurance performance, while *Viscum album coloratum* has been linked to muscle hypertrophy (23). *Astragalus membranaceus (A. membranaceus)* has demonstrated efficacy in enhancing muscle performance in animal studies (24, 25). Clinical study has shown that supplementation with Astragalosides, a major active compound in *A. membranaceus*, can enhance muscle recovery following eccentric exercise-induced damage and reduce inflammatory response (26). *Paeonia japonica* (*P. japonica*) showed anti-cachectic effects in a cancer cachexia animal model (27). In addition, we recently reported the pharmacological effects of equal mixture of *A. membranaceus* and *P. japonica* (named as APX) against sarcopenia in a cancer cachexia mouse model (28). These results suggest that APX may exhibit synergistic effects on muscle hypertrophy in an exercise model. However, its evaluation in muscle mass and strength under healthy conditions has not yet been reported.

Therefore, the present study aimed to investigate the muscle hypertrophic effects of APX and its related mechanisms using a weighted treadmill exercise mouse model.

## 2 Materials and methods

### 2.1 Preparation and fingerprinting of APX

Thirty percent ethanol extracts of *A. membranaceus* and *P. japonica* (specimen: CDA-1 and 2) were obtained from KGC Yebon, a good manufacturing practice (GMP)’ facility (Chung-book, Korea). Briefly, 1 kg of each herb was extracted in 20 L of 30% ethanol at 65°C for 4 h. The extracts were filtered and spray dried. The final yields were 22.2% for CDA-1 and 2. The powders were stored at −55°C, and we used a mixture (APX) of equal amounts of each extract for the present study.

Fingerprinting analysis of APX was conducted using high-performance liquid chromatography. Fifty micrograms of APX and 1 μg of each reference compound (albiflorin, paeoniflorin, and formononetin) were dissolved in 1 mL of 70% methanol and the solution was filtered (0.2 μm). A 20 μL volume of each sample solution was injected into an Agilent 1,200 system, and separation was performed using an Agilent Eclipse C18 column (5 μm, 4.6 × 250 mm, Agilent Technologies, CA, USA). The column was eluted at a flow rate of 0.1 mL/min at 35°C and a wavelength of 230 nm using mobile phases A (0.1% formic acid in H_2_O) and B (acetonitrile). The gradient flows were applied as follows: 0–60 min, 15–70% B; 60–65 min, 70–90% B; and 65–70 min, 90–90% B.

### 2.2 Overview of study design

To assess the muscle hypertrophic effects of APX, we employed both a cell-based *in vitro* assay and an *in vivo* experiment. The *in vitro* assay aimed to investigate APX’s impact on myotube differentiation using C2C12 myoblasts and its associated molecular parameters. Meanwhile, the animal study focused on examining the synergistic muscle hypertrophic effect of APX under weighted exercise conditions. Further details are described below.

### 2.3 C2C12 myoblast culture and myotube differentiation

C2C12 myoblasts were obtained from ATCC (VA, USA) and were incubated in Dulbecco’s modified Eagle’s medium containing 10% fetal bovine serum (WelGENE Inc., Kyeong-book, Korea) at 37°C with 5% CO_2_.

Myogenic differentiation of C2C12 myoblasts was performed in 2% horse serum according to previous documentation (29). In brief, C2C12 myoblasts were seeded in 100-mm dishes (1 × 10^6^ cells/dish) and 6-well plates (1 × 10^5^ cells/well). When C2C12 myoblasts were 70% confluent, the serum was replaced daily with 2% horse serum for 5 days with Dulbecco’s phosphate-buffered saline (as a control), APX (50 μg/mL) or insulin growth factor 1 (IGF1, 10 ng/mL, as a positive control). Differentiated myotubes were used for immunofluorescence staining and western blotting. The treatment concentrations of APX on C2C12 myotubes were determined through a cell viability assay with C2C12 myoblasts (data not shown).

### 2.4 Immunofluorescence staining of C2C12 myotubes

For immunofluorescence staining, C2C12 myotubes were fixed with 4% paraformaldehyde for 2 h at 4°C. After washing with phosphate buffered saline (PBS) twice, the cells were incubated with myosin heavy chain primary antibody (MyH, MAB4470, R&D system, MN, USA) containing 5% chicken serum and 1% Triton X-100 in PBS overnight at 4°C. After washing, the cells were incubated with Alexa Fluor 488-conjugated secondary antibodies (ab150113, 1:400; Abcam, Cambridge, UK) at room temperature (RT). After washing, the cells were incubated with 1 μg/mL diamidino-2-phenylindole dihydrochloride (DAPI; Sigma Aldrich, MO, USA) for 2 min at RT in the dark. All signals in C2C12 myotubes were observed using an IX70 microscope at 200× magnification (Olympus, Tokyo, Japan). The myogenic area was calculated using ImageJ software according to NIH guidelines.^1^

### 2.5 Animals

A total of 15 mice (C57BL/6 N, 16 weeks of age, around 34–37 g, male) were purchased from Daehan Biolink (Choong-book, Korea), and a normal chow diet (Rodent NIH-31 Open Formula Auto) was purchased from Zeigler Co. (PA, USA), which was composed of 72% of carbohydrate, 18% protein and 4% fat (total: 3.97 kcal/g). All mice were housed in a cage maintained at 22 ± 2°C under a 12-h light/12-h dark cycle (5 mice per cage) with free access to food and tap water. After 1 week of adaptation, animal experiments were conducted in accordance with the Guide for the Care and Use of Laboratory Animals prepared by the US National Institutes of Health and the procedures approved by the Institutional Animal Care and Use Committee of Daejeon University (DJUARB2023-001).

### 2.6 Weighted-treadmill exercise mouse model

**Figure 1:**
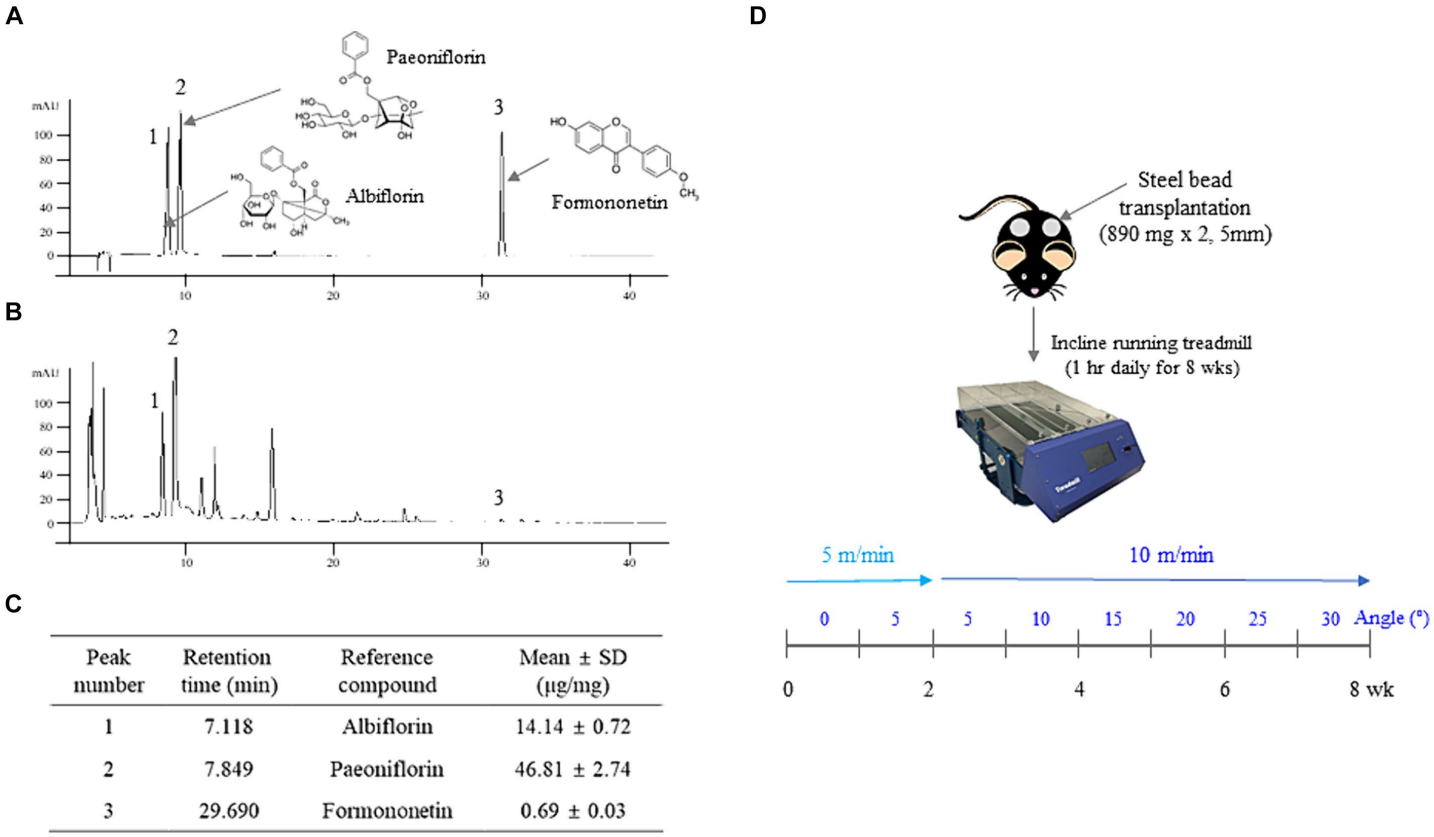
APX Fingerprinting and the treadmill exercise procedure. Three reference compounds **(A)** and APX **(B)** were analyzed by high-performance liquid chromatography (HPLC) and their semiquantification was calculated by a standard curve of reference compounds **(C)**. The mice were transplanted with 2 steel beads (5-mm, 890 mg each) for weighted-exercise. Then, treadmill exercise was conducted for 8 weeks, increasing the angle and speed using an inclined running treadmill **(D)**.

Fifteen mice were randomly allocated into 3 groups (5 mice per group with equal mean weights): control, exercise and exercise/APX. Next, mice were intraperitoneally injected with ketamine (90 mg/kg) and transplanted with 2 steel beads (each 890 mg, 5 mm diameter) at the subcutaneous site of the hind leg for strong resistance. Except for the control group, mice were trained on an inclined treadmill for 8 weeks according to a process that was previously documented (Figure 1D). Mice were orally administered water or APX (50 mg/kg) after finishing exercise for 8 weeks. The dose of APX (100 mg/kg) was selected based on the commonly recommended human dose (500 mg/day per adult). The dose of APX (100 mg/kg) was selected applying body surface area of the human-mouse (12.3-fold) based on the commonly recommended human dose (500 mg/day per 60 kg adult) (30). To minimize observer bias, we ensured that the first author, who managed most of the data, was blinded during the sacrifice of animals and the measurement of samples on the final day.

### 2.7 Food intake and body and tissue weights

Food intake and body weight were measured once a week. One hundred grams of food were added to cages, and the reduction in the amount of food was measured the next week. On the final day of the experiment, the animals were euthanized in a CO_2_ chamber (Jeungdo Bio&Plant, Seoul, Korea). The muscles (gastrocnemius and rectus femoris) and three abdominal fat pads (epididymal, retroperitoneal and visceral) were removed and weighed. The tissues were stored at −80°C in a deep freezer for biochemical analyses and measurement of other parameters.

### 2.8 Measurement of lean body and Fat mass

The lean body and fat masses were measured in the whole body at 0, 4 and 8 weeks using dual-energy X-ray absorptiometry (DXA) InAlyzer (Medikors Co., Seongnam, Korea). Briefly, mice were intraperitoneally injected with ketamine (90 mg/kg), placed on the scanner bed and then scanned according to the instructions for operating the InAlyzer system. The lean body and fat masses were assessed by drawing region of interest (ROI) boxes with the steel bead region removed using image analysis software. The data are presented as an average value of 3 repetitions for each mouse.

### 2.9 Measurement of grip strength

On the day before the final experimental day, grip strength test was performed using a BIO-GT3 tester (BIOSEB, FL, USA) according to the guidelines of International Mouse Phenotyping Consortium (31). In brief, the four limbs of the mouse were placed on a metal grid mounted at a 45° angle and connected to a force transducer. Then, the tail of the mouse was pulled until the mouse let go of the grid. The test was repeated for a total of 3 sets with a 3-min rest time between each set. The data are presented as an average value of 3 repetitions for each mouse.

### 2.10 Histological analyses

On the last day of the experiment, mice were euthanized under anesthesia using a CO2 chamber (Jeungdo Bio&Plant, Seoul, Korea). Both gastrocnemius and rectus femoris muscles were excised, and their weights were recorded. These tissues were preserved in 10% neutral formalin, RNA later Stabilization Solution (Invitrogen, Waltham, MA, USA), or stored at −80°C for subsequent analysis.

For H&E staining, formalin-fixed muscle tissues were sectioned at 10 μm thickness and stained with Mayer’s hematoxylin and eosin (Sigma Aldrich, MO, USA). The stained samples were then mounted on silane-coated slides using Aqueous-Mount (Scytek Laboratories, Logan, UT, USA). All muscle tissues were examined under an IX70 microscope at 200× magnification (Olympus, Tokyo, Japan), and muscle fiber size was determined using ImageJ software following NIH guidelines (see footnote 1).

### 2.11 Measurement of ROS and cytokines

To determine ROS levels in muscles, 100 mg of muscle tissue was homogenized in 1 mL of radioimmunoprecipitation assay (RIPA) buffer. Next, 5 μL of the sample was added to 140 μL of 0.1 M sodium acetate buffer (pH 4.8) in a 96-well microplate. Following an incubation at 37°C for 5 min, 100 μL of the combined mixture solutions of N, N-diethyl-para-phenylenediamine (DEPPD) and ferrous sulfate were added to each sample. The resulting products were measured for absorbance at 505 nm using a microplate reader (Molecular Device Corp., Sunnyvale, CA, USA), and their levels were calculated by referencing a standard curve generated with hydrogen peroxide.

The levels of inflammatory cytokines in muscle tissues were evaluated using commercial enzyme-linked immunosorbent assay (ELISA) kits for tumor necrosis factor (TNF)-α (BD Biosciences, San Jose, CA, USA) and IL-6 (R&D Systems, Minneapolis, MN, USA), according to the manufacturer’s instructions. Protein concentration in the samples was determined using a Pierce BCA Protein Assay Kit (Thermo Fisher Scientific, San Jose, CA, USA). The ELISA results from muscle tissue samples were normalized based on the protein content (pg/mg protein).

### 2.12 Western blot analysis

C2C12 myotubes and muscle tissues were lysed in protein extraction buffer (PRO-PREPTM, iNtRON Biotechnology, Gyeonggi, Korea). The protein concentrations in the samples were measured using Bio-Rad protein assay reagent (Hercules, CA, USA) and were adjusted to the same concentration. Samples were separated by 7 or 12% polyacrylamide gel electrophoresis and transferred to polyvinylidene fluoride membranes. After blocking in 5% skim milk or bovine serum albumin for 1 h, the membranes were probed overnight at 4°C with primary antibodies against MyH, MyoD (MA1-41017, Thermo Fisher Scientific, PA, USA), myogenin (ab1835, Abcam, Cambridge, UK) and α-tubulin (ab7291). Then, the samples were washed three times for 15 min each and incubated for 2 h with HRP-conjugated anti-rabbit or anti-mouse antibodies. Blots were developed using an advanced chemiluminescence kit, and immunoreaction was visualized using UV fluorescence auto exposure in the Fusion Solo S system (VILBER ROURMAT, France). Semiquantitative analysis of protein expression was performed using ImageJ software (NIH).

### 2.13 Statistical analysis

The results are presented as the mean ± standard deviation (SD). Differences among the groups were analyzed using one-way analysis of variance (ANOVA), followed by *post hoc* multiple comparisons for each group using Tukey’s Honestly Significant Difference (HSD) test, performed with Prism version 7.0 (GraphPad, CA, USA). Statistical significance is denoted as follows: *p < 0.05 or **p < 0.01 for control vs. APX or IGF1 in the cell-based assay, and control vs. exercise or exercise plus APX treatment, respectively.

## 3 Results

### 3.1 Fingerprinting analyses of APX

The chemical compositions of APX were detected as albiflorin, paeoniflorin and formononetin at retention times of 7.118, 7.849, and 29.690 min, respectively. The amount of each compound contained in APX was as follows: 14.14 ± 0.72 μg/mg albiflorin, 46.81 ± 2.74 μg/mg paeoniflorin and 0.69 ± 0.03 μg/mg formononetin (Figures 1A–C).

### 3.2 APX promotes myogenesis in C2C12 myotube-differentiation

**Figure 2:**
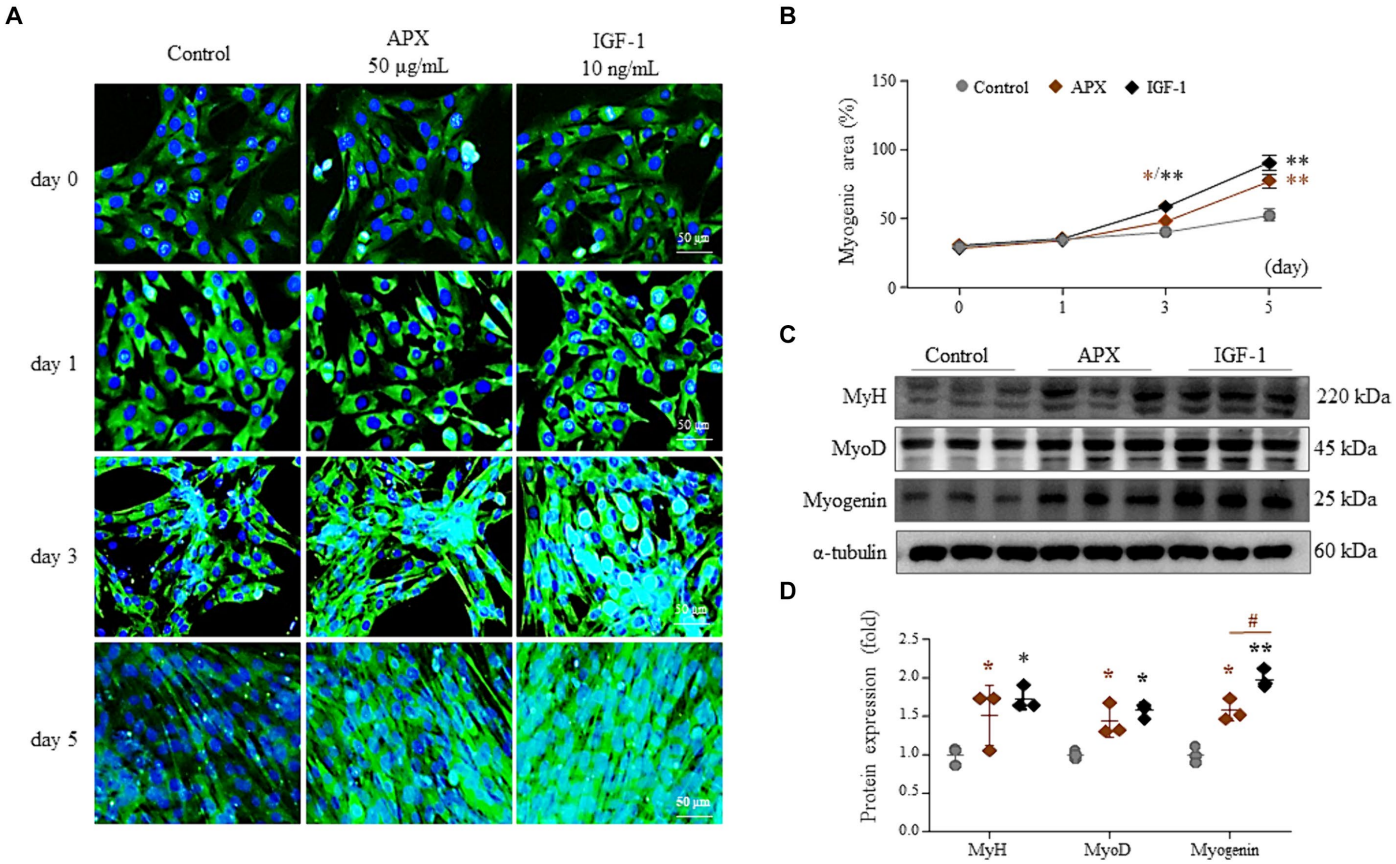
Effects of APX on C2C12 myotube differentiation. C2C12 myoblasts were differentiated by exposure to 2% horse serum with/without APX (50 μg/mL) or IGF (10 ng/mL) for a total of 5 days. Immunofluorescence staining **(A)** was performed 0, 1, 3, and 5 day after 2% horse serum, and the myogenic area was measured using Image J software **(B)**. Western blotting was conducted to determine the protein levels of MyH, MyoD and myogenin on the final day of differentiation **(C)** and their intensities were quantified by Image J **(D)**. For all analyses, statistical significance is presented below: ^*^p < 0.05 or ^**^p < 0.01 for Control vs. APX or IGF1, ^#^*p* < 0.05 for APX vs. IGF1.

Treatment with 2% horse serum for 5 days led to myotube differentiation of 53%. However, the addition of APX or IGF-1 gradually elevated myotube differentiation from 3 days onward to greater levels than that treatment with horse serum alone (*p* < 0.05 or *p* < 0.01). Treatments with APX or IGF1 resulted in a 1.5- to 1.7-fold increase at 5 days compared to treatment with horse serum alone (*p* < 0.01, Figures 2A,B). Treatment with APX or IGF-1 also significantly increased the protein levels of MyH, MyoD and myogenin (*p* < 0.05 or *p* < 0.01, Figures 2C,D).

### 3.3 APX regulated body balance in treadmill exercise mice

**Figure 3:**
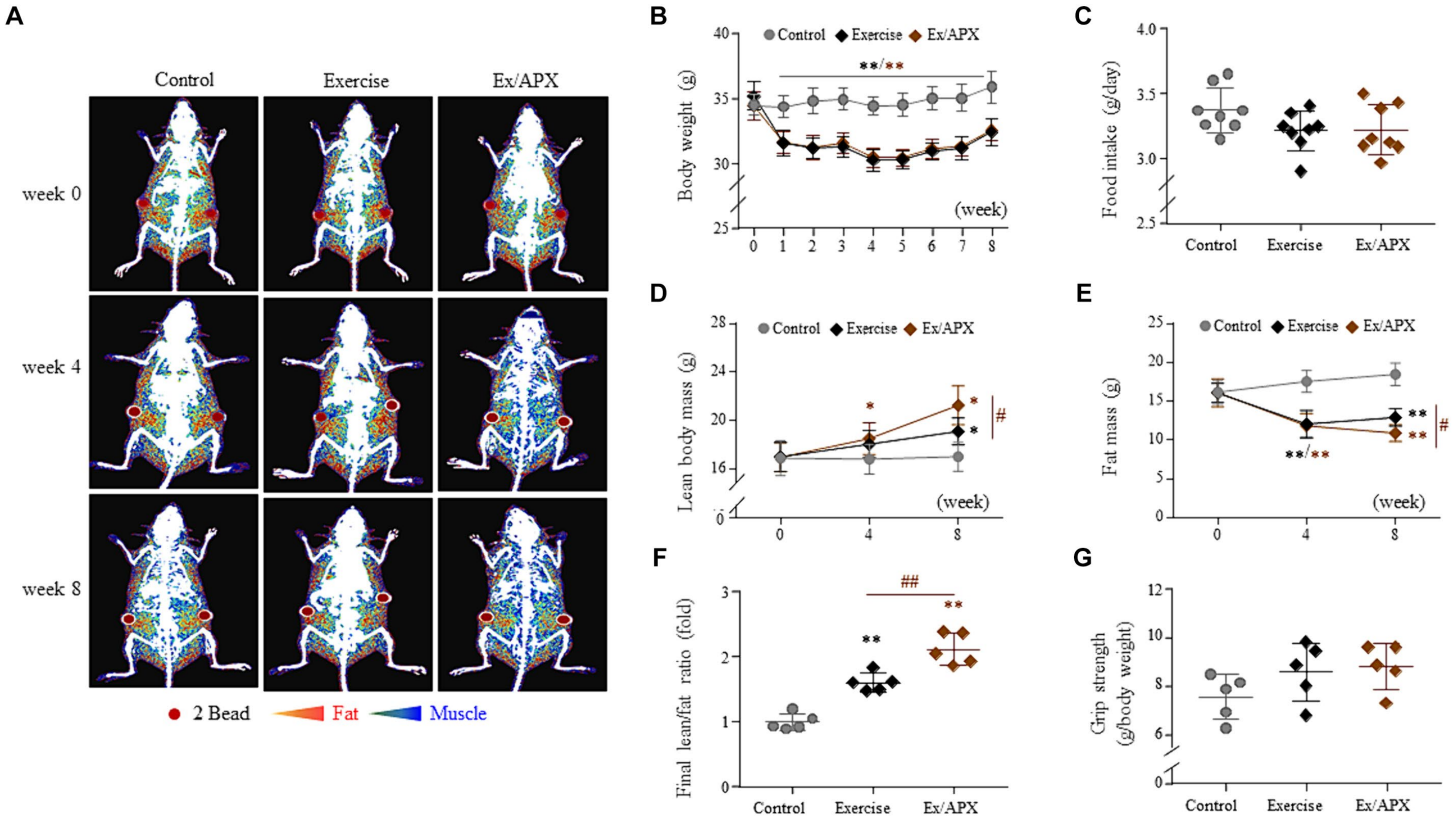
Synergistic effects of APX on the general outcomes in exercised mice. Body weight **(B)** and food intake **(C)** were measured once a week for 8 weeks. At 0, 4, and 8 weeks, the body compositions of mice were visualized using DXA **(A)** and analyzed to measure the lean body mass **(D)** and fat mass **(E)** and their ratios **(F)**. Additionally, grip strength was measured using a BIO-GT3 tester on the final day **(G)**. For all analyses, statistical significance is presented as follow: ^*^p < 0.05 or ^**^p < 0.01 for Control vs. Exercise or Exercise plus APX, ^#^*p* < 0.05 or ^##^*p* < 0.01 for Exercise vs. Exercise plus APX. ^#^*p* < 0.05 or ^##^*p* < 0.01 (control vs. exercise), and ^*^p < 0.05 or ^**^p < 0.01 (control vs. exercise/APX).

Exercise dramatically induced body weight loss from 2 weeks onward and decreased the final body weights approximately 10% compared to the nonexercised group (*p* < 0.01, Figure 3B). Although APX administration did not affect body weight loss, DXA data showed a significantly increased lean body but reduced fat mass in APX mice compared to that in the exercise-only mice (*p* < 0.05, Figures 3A,B,D–F). No significant change was observed in either food intake or grip strength among the three mouse groups (*p* > 0.05, Figures 3C,G).

### 3.4 APX accelerated myogenesis in treadmill-exercised mice

**Figure 4:**
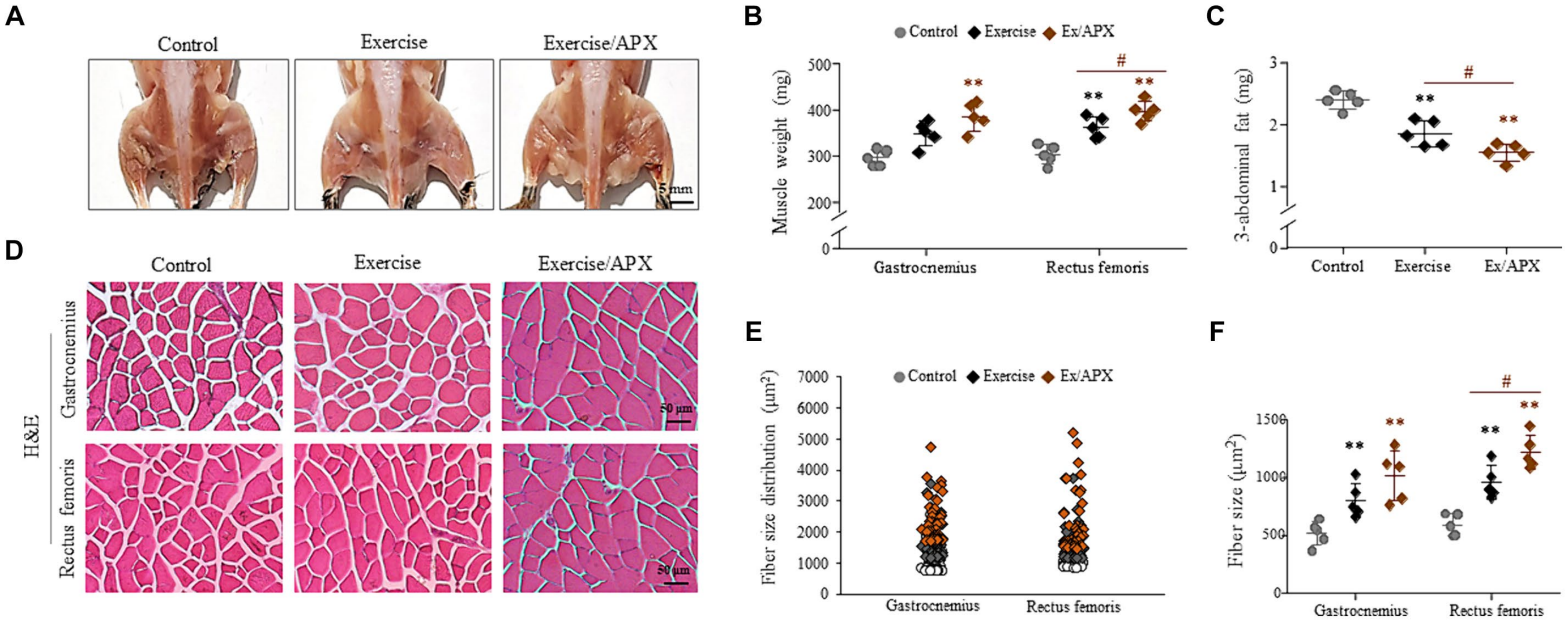
Synergistic effects of APX in muscle tissue. After sacrificing the mice, images of the lower body were obtained **(A)**, and the muscle samples and 3 abdominal fat depots were weighed **(B,C)**. H&E staining was performed in muscle tissue **(D)**, and muscle fiber sizes were measured using Image J software **(E,F)**. For all analyses, statistical significance is presented as follow: ^*^p < 0.05 or ^**^p < 0.01 for Control vs. Exercise or Exercise plus APX, ^#^*p* < 0.05 for Exercise vs. Exercise plus APX.

The 8-week exercise increased the final muscle weight and decreased three abdominal fat depot weights (epididymal, retroperitoneal and visceral). These changes were significantly enhanced by APX administration (*p* < 0.05, Figures 4A–C). Histological analysis supported APX-derived muscle hypertrophic effects (*p* < 0.05, Figures 4D–F), revealing a superior impact on muscle weight and fiber size compared to mice subjected to exercise alone especially in rectus femoris (*p* < 0.05, Figures 4C,F).

### 3.5 APX activated myogenesis-related molecule in treadmill-exercised mice

In the protein assays conducted on the muscles of the gastrocnemius and rectus femoris, three myogenesis-related molecules (MyH, MyoD, and myogenin) showed slight activation following the 8-week exercise regimen. However, APX administration significantly upregulated these molecules compared to the non-exercised group (*p* < 0.05 or *p* < 0.01, Figures 4A,B). These changes were particularly notable in the rectus femoris, demonstrating statistical significance compared to the exercise-only group (*p* < 0.05, Figure 4B).

### 3.6 Effects of APX on ROS and pro-inflammatory cytokines in treadmill-exercised mice

**Figure 5:**
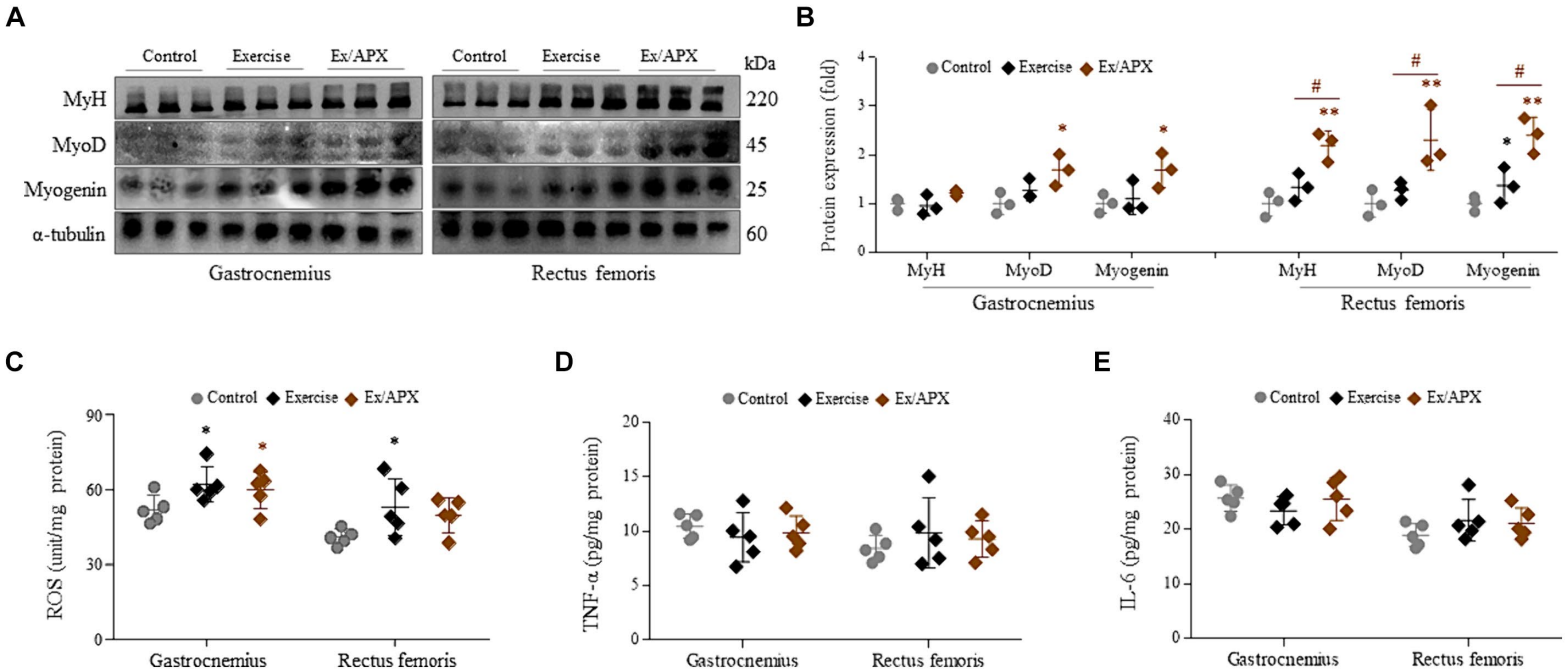
Effects on muscle trophic molecules of APX in muscle tissue. Western blotting was conducted to determine the protein levels of MyH, MyoD and myogenin in muscle tissue **(A)** and their intensities were quantified by Image J **(B)**. The levels of ROS **(C)**, TNF-α **(D)**, and IL-6 **(E)** were assessed in muscle tissue. For all analyses, statistical significance is presented as follow: ^*^p < 0.05 or ^**^p < 0.01 for Control vs. Exercise or Exercise plus APX, ^#^*p* < 0.05 for Exercise vs. Exercise plus APX.

The levels of ROS in the gastrocnemius and rectus femoris showed significant elevation following the 8-week exercise regimen (*p* < 0.05). However, these changes were slightly attenuated by APX administration (*p* > 0.05, Figure 5C). Conversely, the muscular levels of two pro-inflammatory cytokines (TNF-α and IL-6) were not notably altered by the 8-week exercise or APX administration (*p* > 0.05, Figures 5D,E).

## 4 Discussion

Based on our previous results for the anti-cachexia effects of APX (28), we herein explored the muscle-hypertrophic effects using both an exercise-derived animal study and a myotube differentiation cell model. Treadmill exercise protocols are widely used in animal models (32), and weighted-treadmill protocols have been developed using vests or beads for heightened hypertrophy (33, 34). Thus, we adapted an inclined treadmill exercise, which is the most similar method to human exercise, and maximized muscle hypertrophy via transplantation of steel beads (Figure 1D). Many studies recommend treadmill running exercise to protect type 2 diabetes, osteoporosis and sarcopenia in elderly people because it is associated with catabolic activity as well as improvement in metabolic and cardiovascular function and loss of body weight and fat mass in humans (35–37). Chronic treadmill exercise is also strongly suggested to promote anabolic activity leading to muscle hypertrophy (38).

As we expected, in our study, 8 week-exercise rapidly decreased abdominal fat and body weights, but increased lean body mass (Figures 3, 4E). On the other hand, administration of APX significantly accelerated the gain of lean body mass without changing body weight and muscle power compared with the exercise-only group (Figures 3B,D,G). Skeletal muscle is composed of three types of fibers, which are classified based on two criteria: how fast the fibers contract and how the fibers regenerate adenosine triphosphate (ATP) (39). Type 1 fibers contract relatively slowly and use aerobic respiration (oxygen and glucose) to produce ATP for contractions over long periods, in contrast to type 2A fibers, which function in fast contractions using aerobic respiration. Another type 2 fiber (type 2B fibers) perform fast contractions using primarily anaerobic glycolysis (40). Muscle hypertrophy indicates the synthesis of these fibers leading to an increase in muscle mass, which is usually induced under conditions of chronic exercise (41).

The aging process has a marked impact on the structural and functional characteristics of human skeletal muscle, specifically compromising contractile function, which explains the decreased oxidation and glycolytic capacity in type 2 fibers (42, 43). The administration of APX increased the masses of the gastrocnemius and rectus femoris under 8 weeks treadmill exercise (Figures 4A,B,D). The gastrocnemius and rectus femoris muscles, as a components of the leg muscle, predominantly composed of type 2 fibers, and these muscles are specialized for running (44). During exercise, these muscles produce reactive oxygen species and free radicals; however, repeated exercise activates the regeneration of fibers (45). Interestingly, oxidative and inflammatory markers were not significantly different among the three groups of mice (Figures 5C–E). This finding might be the result of muscle adaptation to regular and repeated running exercise. The adaptative pattern of fat-metabolic activity were found, specifically a rapid reduction for the first 4 weeks but a gradual recovery after that (Figures 3B,E), which was in accordance with other findings (46).

To explore the molecular mechanisms underlying the effects of APX on muscle hypertrophy, we examined myosin synthesis and its related molecules in mouse muscles. Myogenesis is a process that involves both producing myoblasts and differentiating them into myotubes (47). In particular, adults are more dependent on myotube differentiation than on the production of myoblasts, in contrast to the growth seen in children (48). Myoblasts, in fact, sequentially differentiate into myocytes and further into myotubes, which is regulated by myogenic transcription factors such as Myf5, MyoD, Mrf4 and myogenin (49). In particular, each MyoD and myogenin play vital roles in differentiation of myoblasts into myocytes and myotubes, respectively, as essential regulators of adult myofiber growth and muscle homeostasis (50, 51). In our study, the protein synthesis of MyoD and myogenin along with an increase in MyH were significantly promoted by APX treatment the in both the muscle tissue of the exercised mice (Figures 5A,B) and in cell-based assays using horse serum-activated C2C12 myoblasts (Figures 2C,D). Although the detailed mechanism is limited, our C2C12-derived results suggested that APX exerted a similar action as IGF1, the most well-known growth factor activating the above two transcription factors (Figures 2A,B) (52).

Maintaining muscle function has steadily been emphasized to sustain a healthy status and prevent aging-related diseases such as diabetes, heart disease, and osteoporosis (53). The above findings support the pharmaceutical potency of APX, especially in muscle hypertrophy and muscle heath. This study, however, has several limitations. These include assessing APX’s synergistic hypertrophic effects with weighted exercise without evaluating the efficacy of APX alone. While our study aimed to emulate the practical approach recommended in clinical fields, involving the recommendation of exercise – a known inducer of muscular hypertrophy - either with or without dietary supplements, this experimental design might introduce uncertainty into the present findings. Another important limitation is the lack of sufficient information regarding the active compounds responsible for its pharmaceutical activity. In present study, the three potential compounds (formononetin, albiflorin, and paeoniflorin) were detected from APX (Figures 1A–C). Formononetin showed anti-muscle atrophic effects in chronic kidney disease mice (54) and myogenic effects via regulation of MyoD and myogenin in C2C12 myotubes (55). In a recent study, anti-muscle atrophic effects of paeoniflorin were reported in ovariectomized and chronic kidney disease mice (56, 57). These evidences strongly support the myogenic effects of APX in present study. However, further verification of active compounds against our models is still needed in the future.

Taken together, our findings provide experimental evidence that a mixture of 30% ethanol extracts of *Astragalus membranaceus* and *Paeonia japonica* has muscle hypertrophic effects, and its underlying mechanisms involve the modulation of the myogenic transcription factors, MyoD and myogenin.

## Data availability statement

The raw data supporting the conclusions of this article will be made available by the authors, without undue reservation.

## Ethics statement

The animal study was approved by Institutional Animal Care and Use Committee of Daejeon University (DJUARB2023-001). The study was conducted in accordance with the local legislation and institutional requirements.

## Author contributions

S-BL: Conceptualization, Data curation, Formal analysis, Investigation, Project administration, Software, Supervision, Validation, Visualization, Writing – original draft. T-WW: Formal analysis, Methodology, Software, Writing – review & editing. D-CB: Formal analysis, Methodology, Software, Writing – review & editing. CS: Conceptualization, Funding acquisition, Project administration, Supervision, Validation, Writing – review & editing.

## Footnotes

^1^http://rsb.info.nih.gov/ij/
